# MODELS: a six-step framework for developing an infectious disease model

**DOI:** 10.1186/s40249-024-01195-3

**Published:** 2024-04-17

**Authors:** Jia Rui, Kangguo Li, Hongjie Wei, Xiaohao Guo, Zeyu Zhao, Yao Wang, Wentao Song, Buasiyamu Abudunaibi, Tianmu Chen

**Affiliations:** 1 State Key Laboratory of Vaccines for Infectious Diseases, Xiang An Biomedicine Laboratory, School of Public Health, Xiamen University, Xiamen city, China; 2 State Key Laboratory of Molecular Vaccinology and Molecular Diagnostics, National Innovation Platform for Industry-Education Integration in Vaccine Research, Xiamen University, Xiamen City, China

**Keywords:** Epidemic models, Model construction, MODELS framework

## Abstract

Since the COVID-19 pandemic began, a plethora of modeling studies related to COVID-19 have been released. While some models stand out due to their innovative approaches, others are flawed in their methodology. To assist novices, frontline healthcare workers, and public health policymakers in navigating the complex landscape of these models, we introduced a structured framework named MODELS. This framework is designed to detail the essential steps and considerations for creating a dependable epidemic model, offering direction to researchers engaged in epidemic modeling endeavors.

**Graphical Abstract:**

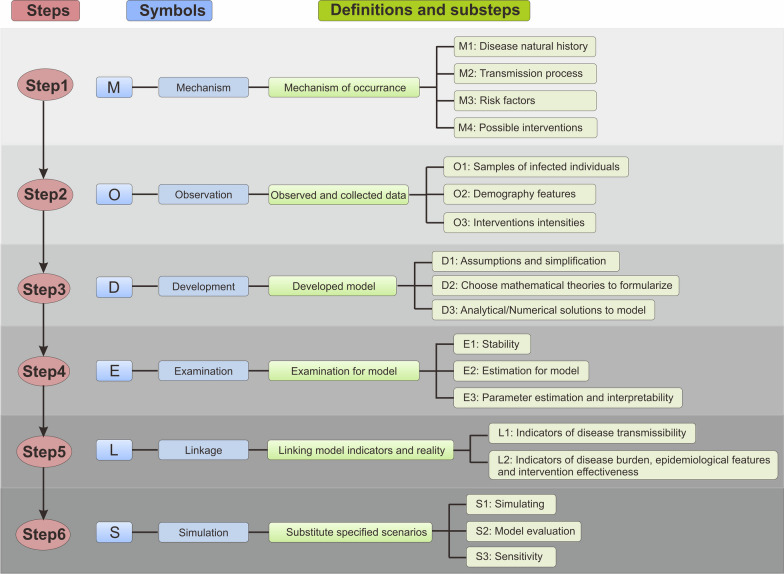

**Supplementary Information:**

The online version contains supplementary material available at 10.1186/s40249-024-01195-3.

## Background

Since the outbreak of the coronavirus disease 2019 (COVID-19) pandemic, numerous COVID-19 modelling studies have been published. Although some proposed models are noticeable and exhibit creative designs, others contain methodological errors. Considering advancing of knowledge regarding disease epidemic characteristics, transmission patterns, control strategies, and the impacts of public health and social measures (PHSMs), researchers have increasingly utilized mathematical language and models to quantitatively elucidate the dynamics of disease spread among hosts, as well as the interplay between etiology and the environment. This endeavor has culminated in the development of theoretical epidemiology, enabling a comprehensive exploration of the effects of diverse preventive and control measures. However, epidemiological models of various diseases are often constrained by inherent limitations arising from the challenges of model selection and construction.

Grappling with the diverse content of these models is challenging for beginners, primary health workers, and public health officials. In this study, we developed a novel framework for developing an infectious disease model called MODELS, comprising six steps: (1) Mechanism of occurrence, (2) Observed and collected data, (3) Developed model, (4) Examination for model, (5) Linking model indicators and reality, and (6) Substitute specified scenarios.

**Fig.1:**
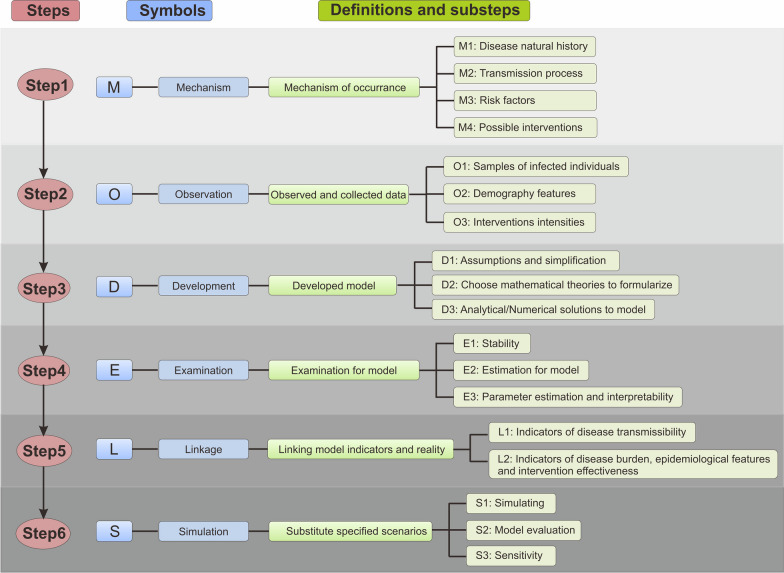
MODELS framework

We also outline the process of model construction (Fig. 1), establish an infectious disease modelling framework, and provide researchers with valuable insights into future modelling endeavors. Our proposed framework provides guidance for researchers interested in epidemic models.

## M: Mechanism of occurrence

The first step, the mechanism of occurrence in infectious diseases, involves a complex interplay of factors that determine the development, transmission, and control of these diseases. Understanding the mechanism of occurrence is fundamental for accurate modelling and prediction of disease dynamics, as well as for developing effective intervention strategies. In this section, we delve into the key components that constitute the mechanism of occurrence, including the natural history of disease, transmission process, risk factors, and possible interventions. During determining the process of mechanism of occurrence in infectious diseases, we often encounter various challenges. This is particularly true in the early stages of a novel infectious disease outbreak, where there tends to be a lack of clear understanding of its natural history. Therefore, it is essential to integrate and continuously update data from clinical, epidemiological, and laboratory studies in order to ensure the reliability of these parameters.

## M1: Disease natural history

The natural history of a disease encompasses its entire trajectory, starting from its onset and progressing through various stages to its outcome without any treatment or intervention [1]. In the first step of modelling, it is necessary to consider whether to develop the study at the individual perspective or at the group perspective. The disease process is characterized by dynamic changes in an individual's status, including susceptible individual, exposed individual, symptomatic or asymptomatic infected individual, and recovered individual. From the group perspective, this means that the population is divided into groups based on their status at different times, these categories have similar transmission characteristics and don’t need to consider differences at the individual level.

When considering the natural history, the key epidemiological characteristics of the infectious disease are considered, including infectivity, pathogenicity, and virulence. It is essential to elucidate the natural history of the disease process by tracking these status updates. The status flowchart varies depending on the specific type of infectious disease.

## M2: Transmission process

Developing a dynamic transmission model requires a comprehensive understanding of the disease, encompassing various aspects such as transmission patterns, incubation periods, infectious periods, and population demographics. Selecting an appropriate modelling approach relies on understanding the primary modes of transmission, such as respiratory droplets, direct contact, and vector-borne transmission through organisms such as mosquitoes.

Transmission dynamic models are based on essential characteristics known as the "three links" (infectious source, transmission route, and susceptible population) and the "two factors" (natural and social factors). These models consider multiple transmission routes, including human-to-human, environmental (e.g., through food or water), and vector-to-human transmission. Additionally, the influence of natural factors, such as environmental conditions like temperature and humidity, on pathogen survival and transmission is considered.

Dynamic transmission models incorporate practical control measures to align with real-world transmission and disease control efforts. These measures encompass both pharmacological interventions, such as antiviral drugs, antibiotics, and vaccines, and non-pharmacological interventions, such as contact tracing, testing and screening, school closures, hand hygiene, social distancing, and mask-wearing. Environmental disinfection, drinking water treatment, and vector control strategies are also considered.

## R3: Risk factors

**Fig. 2:**
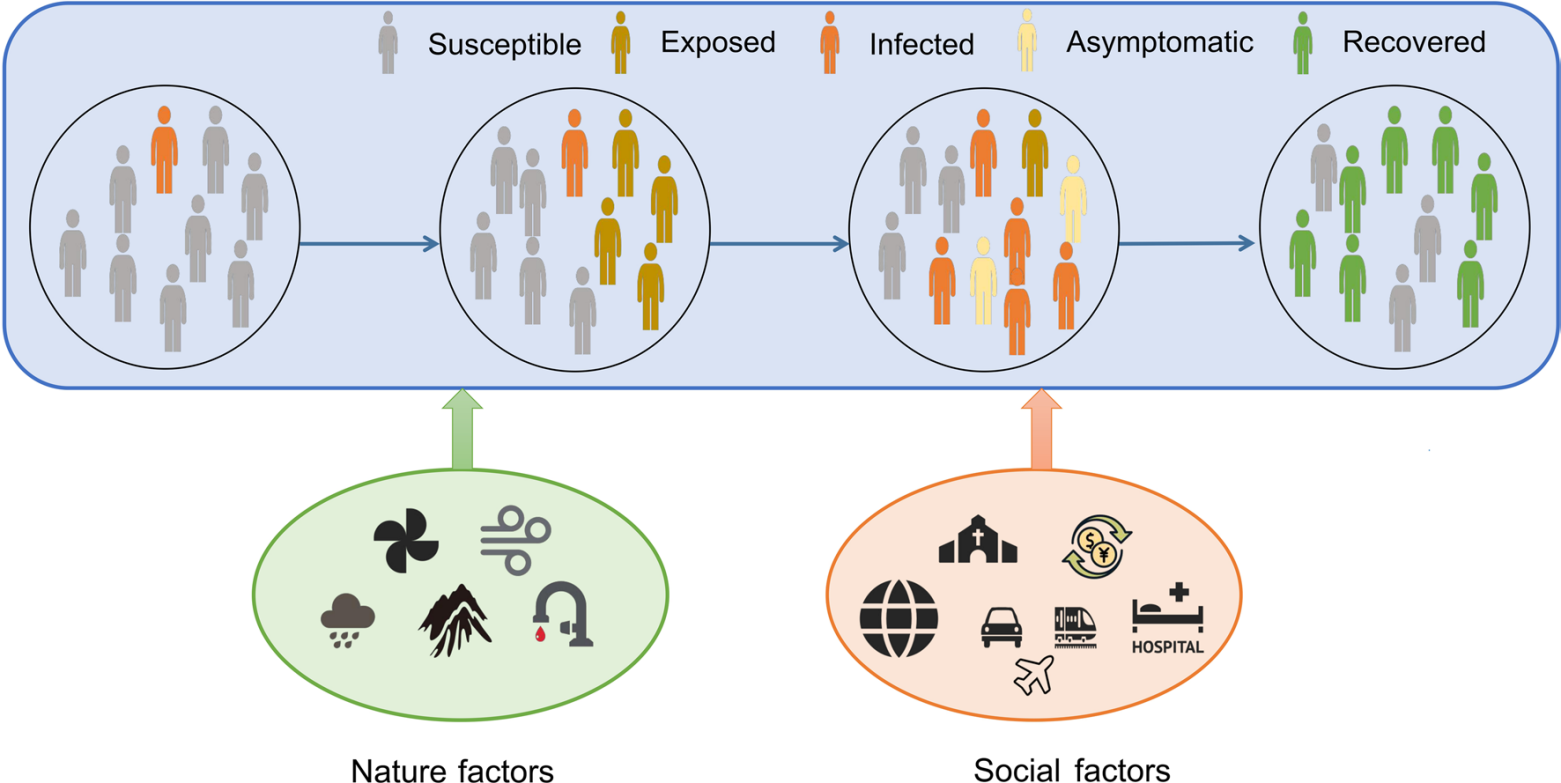
Risk factors on disease transmission

Risk factors play a critical role in the transmission and impact of infectious diseases. By understanding and identifying these factors, we can gain insights into the vulnerability of populations, the severity of disease outcomes, and the potential for disease spread. In this section, we explore two broad categories of risk factors: nature and social factors (Fig. 2).

### M3.1 Nature factors

Nature factors include a range of environmental, geographic, and ecological factors that influence the prevalence and distribution of infectious diseases. For example, meteorological factors, such as temperature, humidity, and rainfall patterns, directly affect the activity and transmission of pathogens. Geographic factors, including terrain, proximity to water bodies, and elevation, can affect the distribution of disease vectors or reservoirs. Ecological factors consider the intricate interactions among pathogens, hosts, and the environment, highlighting the complex dynamics that contribute to disease emergence and persistence.

Geographical factors have a significant impact on disease prevalence. The distribution of diseases and their vectors is influenced by the terrain, proximity to water bodies, and elevation. For example, the geographical distribution of vector organisms varies considerably. Meteorological factors play crucial roles in the transmission dynamics of insect-borne infectious and zoonotic diseases. Temperature directly affects the activity and growth cycles of insect vectors. Furthermore, temperature also has a greater impact on respiratory infectious diseases; lower temperatures during winter, combined with weakened human resistance, tend to result in a higher incidence of respiratory infections such as influenza.

Ecological and meteorological factors significantly contribute to the prevalence of infectious diseases. These factors encompass the intricate interactions between pathogens, hosts, and the environment. Disruptions in ecosystems, such as habitat fragmentation, deforestation, and changes in land use, alter the distribution and abundance of disease vectors and reservoirs, leading to increased contact between humans, wildlife, and vectors. This heightened interaction facilitates the spillover of zoonotic diseases into human populations. The ecological balance within ecosystems plays a crucial role in the amplification or suppression of infectious diseases.

### M3.2: Social factors

Social factors encompass various societal and behavioral aspects that influence the transmission of infectious diseases. These factors include socioeconomic conditions, living standards, healthcare access and infrastructure, educational levels, cultural practices, and population density.

Socioeconomic conditions and living standards significantly affect the disease spread. Access to clean and hygienic living environments free from toxins is essential for reducing the occurrence of certain diseases.

Healthcare access and the level of public health services are critical factors affecting infectious disease outcomes [2]. Improved medical and health conditions coupled with robust public health measures enhance disease prediction, diagnosis, and treatment. Increased vaccine coverage and improved detection systems reduce the incidence of infectious diseases.

Moreover, the social system and speed of government response significantly affect epidemic control. The strict enforcement of importation measures, quarantine protocols, and effective treatment strategies have proven crucial in containing the spread of infectious diseases, as exemplified during the COVID-19 pandemic.

Recognizing the interplay between social factors and infectious diseases is vital for effective disease management and prevention. By understanding the societal context, interventions can be tailored to address specific risk factors and promote behavioral changes. To achieve comprehensive and sustainable disease control, collaboration between PHSMs and environmental factors is essential.

Overall, a comprehensive understanding of the social factors and other epidemiological considerations is crucial for designing and implementing effective strategies to mitigate the impact of infectious diseases and protect public health.

## M4: Possible interventions

**Fig. 3:**
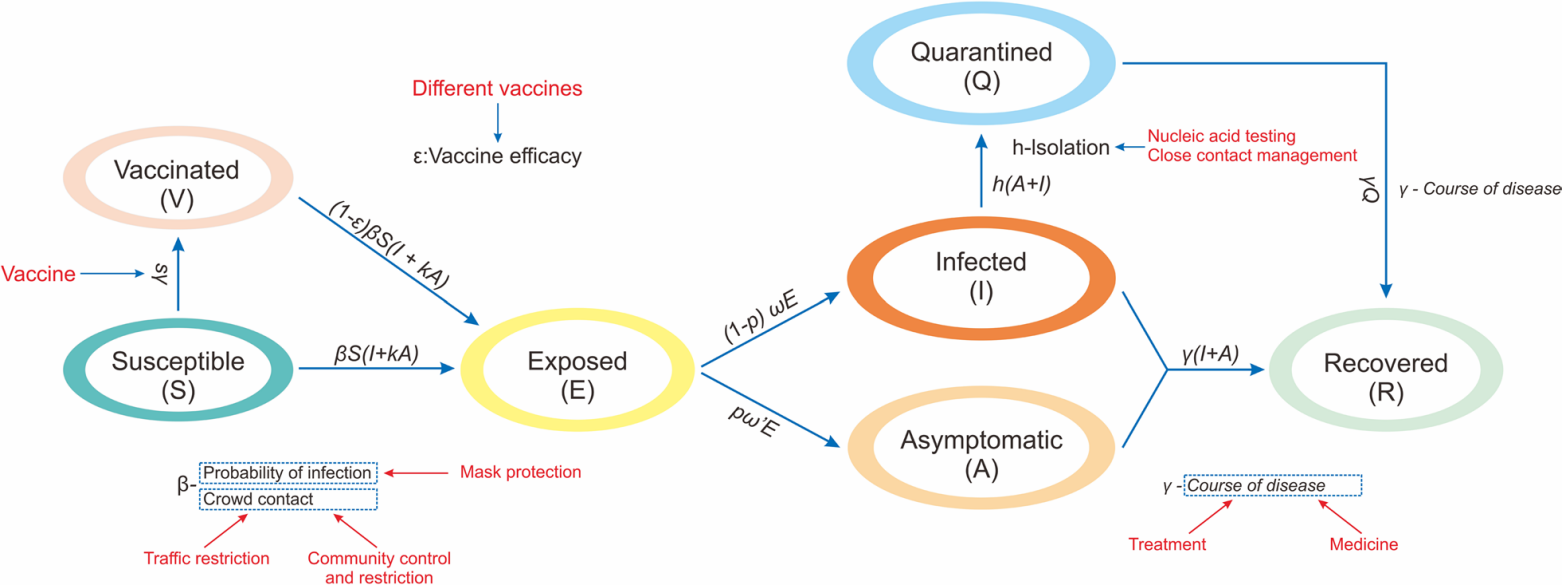
The process by which interventions affect the transmission process of infectious diseases

According to the characteristics of various infectious diseases, integrated interventions are implemented to prevent the continued spread of infectious diseases by targeting the leading links of transmission. Three basic components of the epidemiological process of infectious diseases are targeted (Fig. 3).

### M4.1: Managing sources of infection

The key elements include: (1) timely reporting of infectious diseases; (2) control measures for patients, carriers, and close contacts; (3) control measures for animal sources of infection; (4) measures for environmental contamination of infected sites.

### M4.2: Interrupting transmission routes

Specific measures are employed that are based on the transmission process of the infectious diseases: (1) intestinal infectious diseases: effective management of the disposal of feces and other contaminants and environmental disinfection; (2) respiratory infectious diseases: air disinfection, ventilation, and personal protection (e.g., wearing masks); (3) zoonotic diseases: insecticide and pest control; (4) infectious diseases with complex transmission routes: establishment of comprehensive protective measures to address various transmission patterns.

### M4.3: Safeguarding highly susceptible populations

Primary measures include vaccination, developing an immune barrier, providing preventive medications to people at risk, and taking personal protective measures.

## O: Observed and collected data

Observation and data collection are essential for modelling infectious diseases. These activities help determine the epidemiological characteristics of infectious diseases, such as the rate of virus transmission, incubation period, and mode of transmission, which are essential for the accurate modelling and prediction of disease spread. By analyzing the epidemic data, we can forecast the trajectory and magnitude of future outbreaks, assess the effectiveness of control measures, and optimize disease control strategies. For some researchers with primary data, people who work at the center for disease control and prevention or in hospitals, it is often easier to proceed in this step, and they have an established system for data such as disease or vector surveillance. For researchers who do not have access to primary data, there may be some challenges at the step of obtaining accurate and usable data sources. They may only be able to choose open source databases for their research.

## O1: Samples of infected individuals

Case-specific information is essential for understanding the dynamics of infectious diseases. On-site surveys or historical surveillance data are used to gather data on infected individuals. The stratification of infections based on different dimensions is often necessary.

## O2: Demography features

In our increasingly interconnected world, demographic factors play a significant role in disease transmission. Factors such as urbanization, population aging, travel, and migration contribute to the spread of epidemics. Understanding the links among environmental factors, human health, and disease transmission is crucial. Global climate change, for example, affects the distribution of vector-, food-, and water-borne diseases and interacts with vulnerability factors and disease transmission dynamics. Additionally, health equity is closely tied to economic growth, healthcare resources, and accessibility of educational resources. Gathering demographic data, such as birth rates, death rates, population numbers, and migration patterns, from reliable sources such as the World Health Organization (WHO), World Bank, or national statistical yearbooks, helps inform modelling efforts and assess disease risk.

## O3: Intervention intensities

Incorporating interventions into disease models allows the estimation of the impact of improved diagnostics, new drugs, and different control measures. Data on intervention parameters such as treatment efficacy, diagnostic accuracy, and implementation coverage are typically obtained through a thorough review of the scientific literature and relevant studies. These data help assess the effectiveness and cost-effectiveness of interventions in controlling infectious diseases.

The accuracy and validity of infectious disease models can be enhanced by systematically collecting and analyzing relevant data during the observation and data collection phases. This enables researchers to generate more reliable predictions and develop effective strategies for disease control and prevention. Once the necessary data are collected and observed, the next step is to develop a mathematical model representing the transmission dynamics of the infectious disease.

## D: Developed model

Developing a mathematical model representing the transmission dynamics of infectious diseases is a crucial step in epidemiological research. This model is a powerful tool for simulating and understanding how a disease spreads within a population, enabling the exploration of different scenarios, assessment of intervention strategies, and prediction of future trends. It's important to note that the construction of models should be based on the type of disease, research objectives, and available data. In this section, models are categorized into data-driven models and mechanism-driven models. In  “Choose mathematical theories to formalize”, it is mentioned that different models should be selected based on varying conditions.

## D1: Assumptions and simplification

To select the most appropriate model, researchers start with an existing qualitative understanding of the epidemiological process of the disease and then select it concerning the disease type and the study objectives.

### D1.1: Type of disease

Infectious diseases are diseases that arise when a pathogen infects an organism and can be transmitted from person to person, animal to animal, or animal to human. Many different types of infectious diseases have been observed, each of which can be broadly classified according to its transmission characteristics as gastrointestinal, respiratory, contact, blood, and sexually transmitted diseases, as well as animal- and vector-borne infectious diseases. Depending on the categories to which the disease under study belongs, researchers can choose between a purely human-to-human transmission model or a cross-population transmission model.

### D1.2: Objectives of the study

Models can be used to express the epidemiological process of a disease in symbolic numerical formulas that quantitatively reveal inner laws, and are used for analysis, interpretation, prediction, control, or decision evaluation. Further analytical studies of various types of infectious diseases, specifically disease prediction, estimation of transmission capacity, and evaluation of the effectiveness of interventions, are carried out. For example, when simulating the effects of an intervention, the parameters and links to be evaluated for a single intervention or a combination of interventions must be matched, and the parameters are further supplemented or adjusted to evaluate the effects of intervention [3]. It is often possible to construct a transmission model with single or multiple control measures to simulate epidemic trends with single or combined measures, and thus assess the effectiveness of a particular control measure [3, 4].

## D2: Choose mathematical theories to formularize

We classified mathematical models as either data-driven or mechanism-driven (Table 1). Data-driven models predominantly focus on extracting insights and making predictions from existing datasets, while mechanism-driven models concentrate more on formulating models based on the biological and sociological principles underlying disease transmission. These models hold distinct applicative values in varying contexts. In scenarios characterized by the availability of substantial high-quality data combined with a lack of understanding of the underlying mechanisms, the selection of data-driven models is advisable. Conversely, when there is a comprehensive understanding of the mechanisms involved or examining the effects of various intervention strategies, mechanism-driven models become the preferred choice. In practical applications, it is often beneficial to integrate both types of models, enabling a more holistic understanding and effective response to the challenges posed by infectious diseases.

**Table 1:** Overview of data-driven and mechanism-driven models for epidemic modelling

Data-driven models	
	Time regression model
	Logistic differential equation model
	Chart controlling method
	ARIMA
	Monte Carlo algorithm model
	Grey theory model
	Neural network model
	Others
Mechanism-driven models	
	Ordinary differential equations
	Stochastic individual- or agent-based modelling
	Others

### D2.1: The data-driven model

The data-driven model contains a series of models exploring the relationship between disease occurrence and time, which is an important topic in the mathematical modelling of infectious diseases in China. Common methods include time regression, control graph, time series, autoregressive integrated moving average (ARIMA), Monte Carlo algorithm, grey theoretical, and neural network models.

### D2.2: The mechanism-driven model

The mechanism-driven model is classified by different research object types and parameters, including (1) group and deterministic models, such as transmission dynamics models, and (2) individual models and random models, such as agent-based models, multi-agent systems, and cellular automata.

## D3: Analytical/Numerical solutions to model

Except for highly simple models that can be solved analytically, almost all models are too complicated to find analytical solutions and can be solved numerically, such as by using a computer. In general, the procedure employs model formularization techniques to find solutions for the model. The existence and uniqueness of the model solution are inspected in this step. If a solution does not exist, then the model development process must be re-checked. In some large projects, this step may be called “build a computational model for the model.”

## E: Examination for model

After developing and analyzing a mathematical model of infectious disease transmission, it is crucial to thoroughly examine and evaluate it. This step is essential for assessing the validity and accuracy of the model and identifying potential areas for improvement. Examining the performance of the model can ensure that it aligns with empirical observations and provides meaningful insights into the dynamics of infectious diseases. Ensuring model stability is an essential aspect of working with mathematical or statistical models in the step of examination, particularly when they are applied to complex systems like the dynamics of infectious diseases. This process involves both statistical techniques and comparisons with empirical data.

## E1: Stability

Model stability refers to the degree of consistency in the output of a model when slight variations in the epidemic data are observed [5]. In epidemiological research, models are often used to predict disease transmission trends, assess the effectiveness of interventions, and provide a theoretical basis for public health decision-making. If a model lacks stability, even minor changes in the input data can lead to significant variations in the output, thereby affecting our understanding of disease dynamics and the accuracy of intervention strategies.

## E2: Estimation for model

When a model is developed with a specified formulation using specific knowledge of the mechanism and mathematics, it must be examined before it can be used for prediction, estimation, or other applications. First, it must be determined whether the model is self-consistent; that is, it should not be contrary to existing theories. For example, if a model asserts that “a basic reproduction number less than 2 means the disease will spread over almost the entire population,” then something has gone wrong. Second, the model must be well organized and robust to small amounts of noise and missing data. Such an examination involves a stability analysis of the model equations, and error analysis of the numerical methods used to solve the model numerically. After the behavior of the model is tested analytically or numerically, it still must be confirmed that the model explains the data that are already accumulated and whether it is better than the existing models. In such an analysis, modelers may implement parameter fitting, smoothing, or filtering techniques to estimate the state variables and parameters [6, 7].

## E3: Parameter estimation and interpretability

Parameters can usually be divided into two categories: scenario- and disease-specific. Scenario-specific parameters refer to the differences in transmission from different locations, populations, and times, which are represented by the transmission rate coefficient. The initial values of various variables, such as the number of susceptible persons, infectious sources, and immunized populations in the study area, must be set after parameter estimation. Disease-specific parameters are commonly used in natural history. In infectious disease modelling, the calculation and acquisition of parameters such as incubation period (*ω*), disease duration (*γ*), etc. usually involves the following methods: literature review, epidemiological surveys, and data analysis by descriptive statistics.

### E3.1: Estimation of transmission-specific parameters

Transmission-specific parameters mainly include transmission rate (*β),* population exposure, and probability of infection for a single exposure. Such parameters can be estimated in two ways: through field surveys, such as exposure surveys, and simulations, e.g., the fitting of actual epidemic data.

In the cross-sex model, *β* must be split into the transmission rates between male to male (*β*_*mm*_), male to female (*β*_*mf*_), female to female (*β*_*ff*_), and female to male (*β*_*fm*_). In the model across age groups, *β* must be split into transmission rate between different age groups (*β*_*ij*_) and transmission rate within age groups (*β*_*ii*_). In the case of models that consider contaminants in the environment, the environmental transmission coefficient to the population (*β*_*w*_) also must be considered. In the case of cross-population models, the transmission coefficient (*β*_*a*_) of the animal or vector to the population also must be considered.

### E3.2: Estimation of disease-specific parameters

Disease-specific parameters usually refer to disease natural history parameters, such as *ω*, latency period (*ωʹ*), *γ*, infectious period, proportion of occult infections (*p*), proportion of severe cases (*p*_*s*_), and mortality (*f*). Such parameters are relatively variable among different disease species, and differences in parameters between regions for the same disease are usually less pronounced than those between different disease species. When modeling, such parameters can be obtained through first-hand data in the field or through references as they are more difficult to obtain in the field; sensitivity analysis or uncertainty analysis should be carried out appropriately for parameters from references.

### E3.3: Estimation of intervention-specific parameters

Currently, the main preventive and control measures for infectious diseases include pharmacological (vaccination and medication) and nonpharmacological interventions (isolating patients, wearing masks, increasing social distancing, etc.). The effectiveness of non-pharmacological interventions has been confirmed by multiple studies; they successfully control the prevalence of various diseases through the strict implementation of various public health policies, such as isolating cases, tracing close contacts, and social distancing. The corresponding parameter for isolating cases is increasing the isolation coefficient (*φ*), increasing the social distance is reflected in the population contact degree (*x*), and wearing a mask is reflected in changing the probability of infection with a single-contact infection rate (*p*). The study evaluates the effectiveness of vaccination, mainly including the vaccination rate (*δ*) and the vaccine effect parameters. In terms of medication treatment, studies have evaluated the prevention and control effect of the population; the main parameters include the shortening of disease duration (*γ*), the reduction of patient severe illness rate (*q*), and the reduction of severe case fatality rate (*f*_*c*_).

## L: Linking model indicators and reality

The goal of developing mathematical models for infectious disease transmission is to bridge the gap between theoretical insights and practical applications. Although models provide valuable insights into the dynamics of disease spread, it is crucial to establish a strong link between model indicators and real-world observations. This ensures that the predictions and recommendations of the model are relevant, reliable, and actionable for disease control and prevention. Finding appropriate and accurate indicators based on the scientific questions posed by different studies may be challenging for some of the researchers who are at the beginning of their research work.

## L1: Indicators of disease transmissibility

The basic reproduction number (*R*_0_) is an important indicator of the transmissibility of an infectious disease. *R*_0_ is defined as the number of new cases generated by an infected individual in an otherwise fully susceptible population in the absence of interventions. A greater *R*_0_ value indicates greater transmissibility of the infectious disease [8].

When *R*_0_ < 1, the disease will not cause an epidemic, the number of infections will decrease, and the disease will be gradually eliminated. When *R*_0_ > 1, the disease will cause an epidemic. Thus, *R*_0_ = 1 is the threshold for the transmission of infectious diseases [9].

This definition indicates that the calculation of *R*_0_ requires more stringent conditions, that is, the entire population is susceptible. The proportion of the susceptible population declines gradually as the epidemic progresses or interventions are implemented; at this point, it is no longer appropriate to use *R*_0_ to measure propagation capacity. The effective reproduction number (*R*_*eff,*_) or the time-varying reproduction number (*R*_*t*_) should be applied [10].

## L2: Indicators of disease burden, epidemiological features, and intervention effectiveness

The total attack rate (TAR) is the percentage of cumulative cases of a disease in the total population during an epidemic:

$$\text{TAR = }\frac{\text{Cumulative cases}}{\text{total population }}\times{ 100\%}.$$

The total asymptomatic infection rate (AIR) is the percentage of cumulative asymptomatic infections caused by a disease in the total population during an epidemic:

$$\text{AIR = }\frac{\text{Cumulative asymptomatic infection}}{\text{total population }}\times{ 100\%}.$$

The total infection rate (TIR) is the percentage of cumulative infections of a disease in the total population during an epidemic:

$$\text{TIR = }\frac{\text{Cumulative infections}}{\text{total population }}\times{ 100\%}.$$

Thus, TIR = TAR + AIR.

Duration of outbreak (DO) is the time interval from the start of transmission of the infectious disease to the end of the outbreak [1]. DO can be defined in two ways: the time interval from the first case onset to the last case onset, that is, calculated from the epidemic curve; and the time interval from the first case onset to the last case recovery. It is calculated as follows:

$$DO= {t}_{2} - {t}_{1}.$$

where *t*_1_ is the date of onset of the first case and *t*_2_ is the date of onset or recovery of the last case.

Peak incidence is the maximum incidence or number of infectious diseases at the shortest wcalculated time (e.g., day or week) during an epidemic.

The time of peak incidence is the specific time (e.g., date) at which the peak incidence of a disease occurs during an epidemic.

The severity rate is the proportion of severe cases in the total cases and is one of the most important indicators of virulence.

Mortality rate indicates the proportion of deaths due to a disease among patients with that disease in a certain period and indicates the risk of death for patients with that disease [1]:

$${\text{Mortality rate}} = \frac{\text{deaths due to a disease in a certain period}}{\text{total number of cases }}\times { 100\%}.$$

Secondary attack rate (SAR), also known as secondary incidence rate, is an important indicator used to measure the spread of an infectious disease. It is the percentage of susceptible contacts who develop a disease between the shortest and longest incubation periods for certain infectious diseases as a percentage of the total number of all susceptible persons:

$${\text{SAR}} = \frac{\text{Number of cases among susceptible contacts during the incubation period}}{\text{Total number of susceptible contacts}}\times{ 100\%}.$$

## S: Substitute specified scenarios

In infectious disease modelling, the ability to substitute specified scenarios is a fundamental step in bridging theoretical insights with practical applications. By simulating and assessing specific scenarios, researchers can gain a comprehensive understanding of the potential outcomes of various interventions and policy measures.

## S1: Simulating

Building on the groundwork laid out in the previous five steps, the next crucial phase involves running the infectious disease model using computational methods to simulate various disease transmission scenarios. Different diseases exhibit unique modes of transmission – airborne, vector-borne, or direct contact – each necessitating tailored modelling approaches. For instance, models for airborne diseases like influenza might emphasize social interactions and mobility patterns, while those for vector-borne diseases such as malaria need to factor in environmental influences and vector population dynamics. Additionally, the variability in transmission rates, incubation, and infectious periods across diseases necessitates the incorporation of these differences in scenario planning, possibly through simulations that vary contact rates or the duration of infectious periods. Population settings also play a critical role; factors like population density, age distribution, and healthcare access profoundly impact disease spread. High-density areas might require scenarios accounting for overcrowding, whereas rural areas might focus on healthcare accessibility. Cultural and behavioral aspects, such as social gathering prevalence or attitudes towards vaccination and public health practices, alongside economic factors and resource availability, are pivotal in shaping scenario development, especially in lower-income settings with limited health infrastructure. The specificity of scenarios is equally important; they must be relevant to both the disease and its context. For highly infectious diseases like COVID-19, scenarios could range from lockdown measures to mask mandates, while for Human Immunodeficiency Virus/AcquiredImmune Deficiency Syndrome (HIV/AIDS), the focus might be on awareness campaigns or antiretroviral therapy coverage. Moreover, dynamic adaptation of scenarios is essential, responding to evolving disease patterns or new data, like emerging virus variants. In this context, collaboration with public health experts and epidemiologists who have insights into local conditions and disease specifics is invaluable, enhancing both the relevance and effectiveness of the proposed scenarios in model stability assessment.

Researchers can choose to either develop their custom model code or utilize pre-existing packages specifically designed for infectious disease modelling, such as the SimInf and EpiModel packages in R (https://www.r-project.org/), or the epydemic and Eir packages in Python (https://www.python.org/).

Using simulation, researchers can explore the dynamic behavior of the disease under different conditions and interventions. The model generates predictions and projections by the input of specific parameters and variables, thereby providing valuable insights into the potential outcomes of various interventions and policy measures. Simulations enable the assessment of the effectiveness of different control strategies and the evaluation of the impact of preventive measures on disease transmission.

## S2: Model evaluation

Model fitting methods typically include the least squares estimation (LSE), maximum likelihood estimation (MLE), root mean square estimation (RMSE), akaike information criterion (AIC), and bayesian information criterion (BIC). For differential equation models, an algorithm that uses an adaptive step selection strategy, along with the fourth-order Runge–Kutta method with equidistant nodes as the discretization method, is a common algorithm for solving initial-value problems for ordinary differential equations (Table 2).

**Table 2:** Model evaluation for different models

Model type	Key considerations	Assessment techniques
Data-driven models	Focus on learning from and predicting based on existing datasets	MSE, RMSE, *R*^2^, AIC, BIC, historical fitting, predictive validation, sensitivity analysis
Mechanism-driven models	Emphasize modelling based on biological and sociological principles of disease transmission	*R*^2^, AIC, BIC, predictive validation, sensitivity analysis, scenario analysis, expert review

Further goodness-of-fit tests are required to determine whether the differences between the model results and actual data are statistically significant; the goodness-of-fit tests used include the chi-square test. The coefficient of determination (*R*^2^) can also be calculated and tested for statistical significance. Cox regression can be used to analyze the vaccine effects to determine the time of entry into the group and the time to the endpoint. Methods such as multiple regression analyses and generalized linear models are often used to reconcile confounding factors when analyzing influences. Commonly used software includes SPSS (https://www.ibm.com/spss), SAS (https://www.sas.com/), R (https://www.r-project.org/), Python (https://www.python.org/), MATLAB (https://www.mathworks.com/products/matlab.html), and Berkeley Madonna (https://berkeley-madonna.myshopify.com/).

If the model evaluation results are unsatisfactory, it is necessary to revisit Step 3 and reevaluate the model assumptions and construction. This iterative process ensures that the model aligns with real-world observations and produces reliable and accurate predictions. Once the model evaluation results meet the desired criteria, researchers can proceed with the infectious disease modelling process.

## S3: Sensitivity

Parameter sensitivity refers to the degree of influence the model parameters exert on the model output. In epidemiological research, a sensitivity analysis of parameters is used to assess how changes in specific parameters affect a model's results. By altering the model parameters, researchers can understand the contribution of each parameter to the outcome, allowing the modelers to optimize the model and provide more accurate predictions.

The “knock-out” simulation is derived from knock-out technology, an experimental technique used in genetics in which a normal gene is replaced by a defective gene at an identical chromosomal locus, the normal gene thereby being "knocked out" by the defective gene. In modelling studies, the simulation process sets a parameter to zero and estimates its contribution by counting the number of reduced cases or the total incidence rate. For example, in the SEIARW model, the contribution of environmentally mediated afferents is explored by setting *β*_*w*_ to 0 and reflecting its role by counting the number of cases reduced.

The difference between model stability and parameter sensitivity lies in their respective focuses. Model stability concerns the impact of slight variations in input data on the output of the model, whereas parameter sensitivity focuses on the influence of changes in model parameters on the output. Although both concepts involve model stability and reliability, model stability primarily addresses the overall stability of the model, whereas parameter sensitivity examines the impact of individual parameters. In epidemiology, both model stability and parameter sensitivity analyses play crucial roles in understanding and improving the accuracy of epidemiological models.

## Conclusions

The MODELS framework offers a systematic and comprehensive approach to develop infectious disease models. By emphasizing the importance of data collection, model formulation, consideration of social and ecological factors, and model evaluation, this framework provides a roadmap for generating reliable and actionable models. Additional file 1 is a case study based on the MODELS framework. By following the MODELS framework, researchers and policymakers can enhance their understanding of disease dynamics and make informed decisions to effectively control and prevent infectious diseases. Ultimately, this framework will contribute to global efforts to mitigate the impact of infectious diseases.

### Supplementary Information

**Additional file 1: Figure S1.** Flowchart of the susceptible-infected-recovered (SIR) model. **Figure S2.** Cumulative cases of EVD. **Figure S3.** Daily incidence of EVD. **Figure S4.** Simulated cumulative cases of EVD for different basic reproduction numbers and recovery rate.

**Additional file 2. **Example data.

**Additional file 3. **Figure drawing R code.

**Additional file 4. **Sensitivity analysis R code.

## Data Availability

All data and materials are from cited references.

## Abbreviations

COVID-19: Coronavirus disease 2019
WHO: World Health Organization
HIV: Human Immunodeficiency Virus
AIDS: AcquiredImmune Deficiency Syndrome
PHSMs: Public health and social measures
ARIMA: Autoregressive integrated moving average
*R*_0_: Basic reproduction number
*R*_*eff*_: Effective reproduction number
*R*_*t*_: Time-varying reproduction number
TAR: Total attack rate
AIR: Asymptomatic infection rate
TIR: Total infection rate
DO: Duration of outbreak
SAR: Secondary attack rate
LSE: Least squares estimation
MLE: Maximum likelihood estimation
RMSE: Root mean square estimation
AIC: Akaike information criterion
BIC: Bayesian information criterion
*R*^2^: Coefficient of determination
