# Supplementary material for: MODELS: a six-step framework for developing an infectious disease model
Source: Infect Dis Poverty. 2024 Apr 17;13:30. doi: 10.1186/s40249-024-01195-3 (PMC11022334; doi:10.1186/s40249-024-01195-3)
Supplement: Supplementary file 1 — Additional file 1: Figure S1. Flowchart of the susceptible-infected-recovered (SIR) model. Figure S2. Cumulative cases of EVD. Figure S3. Daily incidence of EVD. Figure S4. Simulated cumulative cases of EVD for different basic reproduction numbers and recovery rate. [file 40249_2024_1195_MOESM1_ESM.docx]

# Additional file 1: Appendix

# MODELS: A six-step framework for developing an infectious disease model

The aim of this appendix is to present a thorough collection of additional information that bolsters and elucidates the concepts, data, and analyses presented in the main text of our epidemiology paper. Ebola Virus Disease (EVD) will be used as an example. This appendix encompasses a diverse range of detailed data and explanatory content, which explores the natural history of EVD, the mechanisms behind its transmission, the contributing risk factors, and the imperative intervention strategies necessary for its control.

## M: Mechanism of occurrence

Understanding the epidemiology of Ebola virus disease (EVD) is essential for effective outbreak response and management. The natural history of the disease, transmission dynamics, and associated risk factors are critical components that influence the spread and impact of EVD within affected populations.

### M1: Disease natural history

The course of EVD following exposure is marked by an incubation period, during which there are no symptoms but the virus is incubating within the host. This period has been observed to last an average of 6.22 ± 1.57 days^1^. Following the incubation period, the onset of symptoms marks the beginning of the infectious period, which continues until recovery or death, lasting approximately 9.40 ± 5.50 days^1^.

### M2: Transmission process

Transmission of EVD is primarily through direct contact with the bodily fluids of infected individuals, including blood, secretions, and organs, or indirect contact with surfaces and materials contaminated with these fluids. Secondary transmission routes, such as through the semen of recovered patients or breast milk, present additional challenges for containment efforts. The basic reproduction number (R0) for EVD, an indicator of the contagiousness of the virus, typically ranges between 1.5 and 2.5 in the absence of interventions^2^. This variability underscores the need for context-specific strategies to reduce transmission.

### M3: Risk factors

#### M3.1 Nature factors

Geographical proximity to dense rainforests, where fruit bats, the natural reservoirs of the Ebola virus, reside, may elevate the risk of zoonotic transmission events. However, the focus of this study is on human-to-human transmission, which is the primary concern during outbreaks.

#### M3.2 Social factors

Social behaviors and cultural practices play a significant role in the spread of EVD. Traditional funeral rites involving contact with the deceased can accelerate virus transmission among communities. Furthermore, healthcare settings lacking stringent infection control are hotspots for the amplification of EVD spread.

### M4: Possible interventions

Effective management of EVD is multifaceted, involving several critical strategies that are pivotal in controlling the spread of the virus. One crucial aspect is the use of Ebola Treatment Centers (ETCs), which play a pivotal role in the isolation and treatment of patients, thereby reducing the transmission of the virus within communities. ETCs are equipped with specialized staff and facilities designed to provide supportive care to those affected and to contain the outbreak^3^. Another key strategy is contact tracing, which involves the systematic identification and monitoring of all individuals who have had contact with an infected person. This surveillance, maintained over the virus's maximum incubation period of 21 days, is essential for the early detection of new cases and prompt intervention^4^. Safe burial practices are also integral to controlling EVD spread^5^. Traditional burial practices can facilitate transmission, so ensuring that burials are conducted in a safe and dignified manner is critical to mitigating further infections. Lastly, community engagement is vital for the success of these strategies. It encompasses educating communities about the risk factors associated with EVD and actively working to reduce the stigma associated with the disease^6^.

## O: Observed and collected data

### O1: Samples of infected individuals

Detailed case report data is typically the most desirable for comprehensive analysis. However, in cases where such data is unavailable, utilizing publicly available information becomes an alternative approach. In this study, due to the unavailability of detailed case report data, publicly available historical data from the internet was utilized as an illustrative example. Specifically, data from the Western African Ebola virus epidemic (2013-2016), the most extensive outbreak of EVD in history, was utilized. The outbreak significantly impacted Guinea, Liberia, and Sierra Leone, causing substantial loss of life and socioeconomic disruption in the region. For the purpose of this study, Guinea's data was utilized as a representative example (Supplementary Appendix 2)^7^.

### O2: Demography features

Accurate population numbers are crucial for infectious disease modeling. In this study, the total population of Guinea during the period from 2013 to 2016 was obtained from the World Bank website. The population figures for Guinea during this period were as follows: 11,055,430 (2013), 11,333,365 (2014), 11,625,998 (2015), and 11,930,985 (2016)^8^.

### O3: Intervention intensities

The Ebola epidemic is believed to have originated as a small outbreak in the Guéckédou prefecture of Guinea in late 2013. By March 2014, the number of cases had escalated, spreading to the capital city, Conakry. In response to the escalating crisis, collaborative efforts were initiated between the Centers for Disease Control and Prevention (CDC), the World Health Organization (WHO), and the Guinean government. CDC teams were deployed to Guinea in March, and their presence continued until April, when the outbreak appeared to be subsiding^9^. For the purpose of this study, due to limited availability of intervention intensity data, we categorized the outbreak into two distinct stages: the period before April 2014 and the period after April 2014. This division allows for an examination of potential variations in outbreak dynamics and intervention effectiveness during these distinct phases.

## D: Developed model

### D1: Assumptions and simplification

#### D1.1 Type of disease

In this appendix, we focus on the epidemiology of EVD, an infectious disease caused by the Ebola virus. EVD is primarily transmitted through direct contact with infected individuals' bodily fluids. For the purposes of modeling and analysis, we assume that the transmission of EVD occurs solely through human-to-human contact. This simplification allows us to focus on understanding the dynamics of the disease within the human population..

#### D1.2 Objectives of the study

The main objective of this study is to develop a transmission model to analyze and predict the spread of EVD, assess the effectiveness of interventions, and evaluate various control measures. Through the use of mathematical and computational models, we aim to quantify the impact of different interventions on the epidemic trends of EVD. By simulating the effects of single or combined interventions, we can assess their effectiveness in controlling the spread of the disease. This analysis will aid in informing public health decision-making and intervention strategies.

### D2: Choose mathematical theories to formularize

To formulate the mathematical model for EVD transmission, we focus on deterministic models that capture the dynamics of disease transmission at a population level. Deterministic models utilize differential equations to represent the flow of individuals between susceptible, infected, and recovered states (Figure S1). These models incorporate relevant epidemiological parameters, such as transmission rates and recovery rates, to provide insights into the overall dynamics of EVD transmission. By using deterministic models, we aim to develop a comprehensive understanding of the population-level dynamics of EVD transmission. This approach allows us to analyze the impact of various control measures and interventions on the spread of the disease.


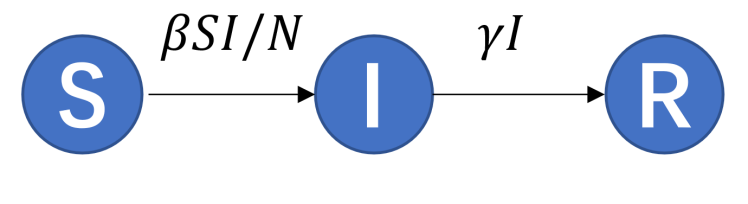


Figure S1: Flowchart of the susceptible-infected-recovered (SIR) model.

### D3: Analytical/numerical solutions to model

To elucidate the dynamics of EVD transmission within the population, we solve the system of differential equations that constitute the Susceptible-Infected-Recovered (SIR) model. The model is based on the following equations:

$$\frac{dS}{dt}=-\frac{\beta SI}{N}$$

$$\frac{dI}{dt}=\frac{\beta SI}{N}-\gamma I$$

$$\frac{dR}{dt}=\gamma I$$

Where ($S$) is the number of susceptible individuals, ($I$) is the number of infected individuals, ($R$) is the number of recovered individuals, ($N$) is the total population size, ($\beta$) is the transmission rate, and ($\gamma$) is the recovery rate. Analytical solutions to these equations can provide insights into the thresholds and conditions necessary for an outbreak to occur or subside. However, due to the complexity of the model and the non-linearity of the equations, exact analytical solutions are often not feasible. Therefore, we employ numerical methods to approximate the solutions. Numerical solutions are obtained through discretization of the equations using methods such as Euler's method, Runge-Kutta methods, or more advanced adaptive algorithms. These numerical techniques allow us to simulate the progression of the epidemic over time, given specific initial conditions and parameter values. The resulting numerical solutions provide a temporal profile of the epidemic, showing how the numbers of susceptible, infected, and recovered individuals change over time. This enables us to predict the peak of the epidemic, the total number of infections, and the duration of the outbreak.

## E: Examination

After developing and analyzing a mathematical model of infectious disease transmission, it is crucial to thoroughly examine and evaluate it. This step is essential for assessing the validity and accuracy of the model and identifying potential areas for improvement. Examining the performance of the model can ensure that it aligns with empirical observations and provides meaningful insights into the dynamics of infectious diseases.

### E1: Stability

Model stability refers to the degree of consistency in the output of a model when slight variations in the epidemic data are observed^10^. In epidemiological research, models are often used to predict disease transmission patterns, assess the effectiveness of interventions, and inform public health decisions. If a model lacks stability, even minor changes in the input data can lead to significant variations in the output, thereby affecting our understanding of disease dynamics and the accuracy of intervention strategies.

### E2: Model estimation

When a model is developed with a specified formulation using specific knowledge of the mechanism and mathematics, it must be examined before it can be used for prediction, estimation, or other applications. First, it must be determined whether the model is self-consistent; that is, it should not be contrary to existing theories. For example, if a model asserts that “a basic reproduction number less than 2 means the disease will spread over almost the entire population,” then something has gone wrong. Second, the model must be well organized and robust to small amounts of noise and missing data. Such an examination involves a stability analysis of the model equations, sensitivity analysis of the parameters, and error analysis of the numerical methods used to solve the model numerically. After the behavior of the model is tested analytically or numerically, it still must be confirmed that the model explains the data that are already accumulated and whether it is better than the existing models. In such an analysis, modelers may implement parameter fitting, smoothing, or filtering techniques to estimate the state variables and parameters^11 12^.

### E3: Parameter estimation and interpretability

#### E3.1: Estimation of transmission-specific parameters

In order to analyze the dynamics of EVD transmission, it is crucial to estimate transmission-specific parameters, which include the transmission rate ($\beta$) and the recovery rate ($\gamma$). These parameters can be estimated using available epidemiological data and the mathematical model. The basic reproductive number ($R_{0}$) is a key parameter in epidemiology. It represents the average number of secondary infections produced by a single infected individual in a fully susceptible population^13^. Using the SIR model, R0 can be calculated as follows:

$$R_{0}=\frac{\beta\frac{SI}{N}}{\gamma I}=\frac{\beta S}{\gamma N}$$

Transmission rate ($\beta$) represents the probability of transmission per contact between a susceptible and an infected individual. It can be estimated using the basic reproductive number and the recovery rate:

$$\beta=\frac{{\gamma NR}_{0}}{S}$$

#### E3.2: Estimation of disease-specific parameters

Disease-specific parameters commonly pertain to the natural history of the disease. In the case of EVD, these parameters are generally centered around the incubation period and the infectious period, as they play a crucial role in comprehending the timing of disease advancement and transmission potential. In this appendix, we have included disease-specific parameters for EVD in the following manner: Drawing from existing literature, we have adopted an average incubation period of 6.22 days, with a standard deviation of 1.57 days, and an average infectious period of 9.40 days, with a standard deviation of 5.50 days^1^.

## L: Linking model indicators and reality

### L1: Indicators of disease transmissibility

For Ebola Virus Disease (EVD), the *R*_0_ is typically estimated to range between 1.5 and 2.5 in the absence of any interventions, according to published literature^2^.

### L2: Indicators of disease burden, epidemiological features, and intervention effectiveness

To effectively link our model to real-world implications, we consider several key indicators: total attack rate (TAR) and peak incidence. These indicators are utilized to evaluate the epidemiological features of EVD and the effectiveness of various interventions. By analyzing the changes in TAR and peak incidence in response to different control strategies, we can infer the efficacy of interventions and guide public health policies.

## S: Substitute specified scenarios

### S1: Simulating

To simulate the epidemic model of EVD, we utilized the R software environment (version 4.3.1, R Core Team, Vienna, Austria). Within R, the 'deSolve' package was employed to solve the system of ordinary differential equations representing the dynamics of the EVD transmission model. All code is availability at Appendix 2. In the absence of any interventions, our model predicts that EVD has the potential to infect the 58.29% population of Guinea, with the peak incidence projected to occur in February 2015. These results underscore the critical importance of timely and effective intervention measures to prevent widespread transmission and control the outbreak (Figure S2, Figure S3).


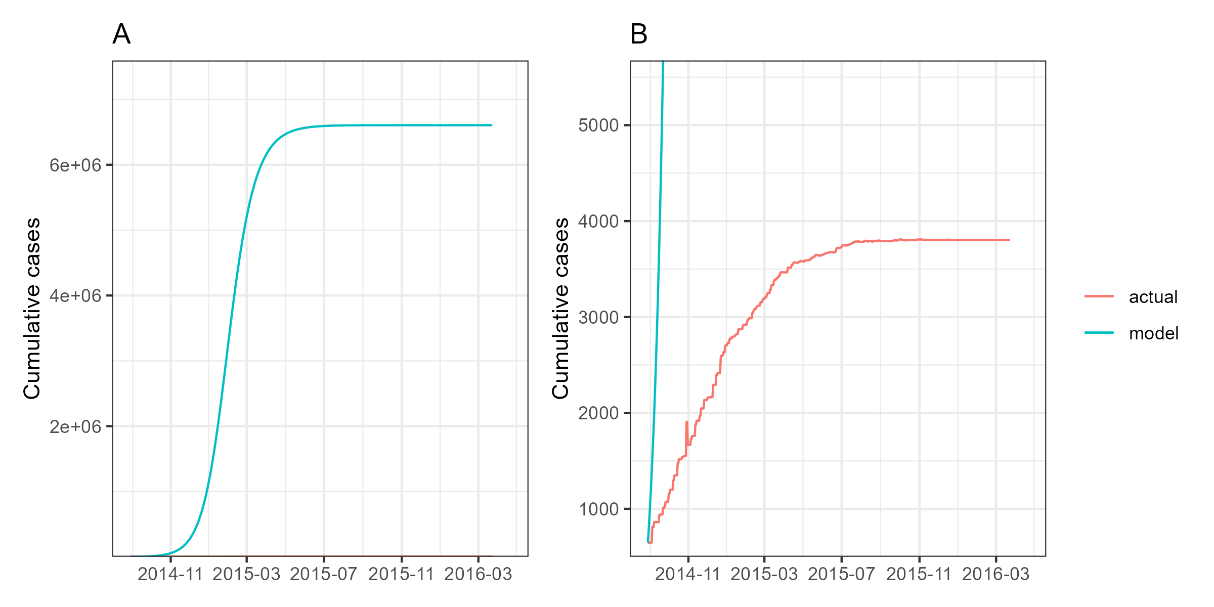


Figure S2: Cumulative cases of EVD.


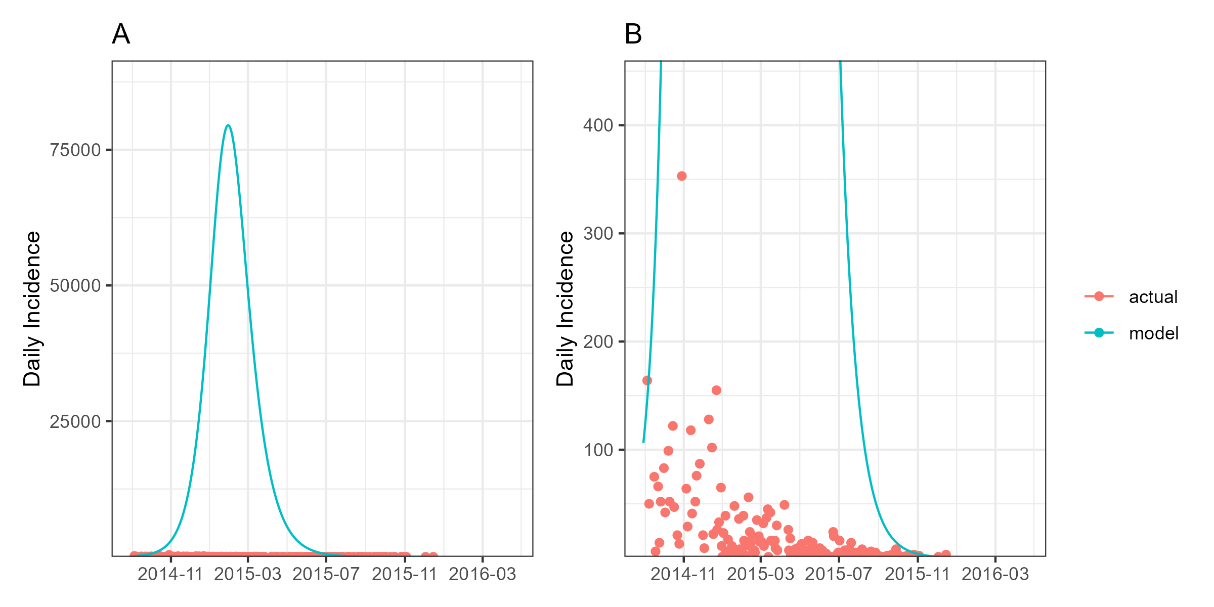


Figure S3: Daily incidence of EVD.

### S2: Model evaluation

This section is not applicable within the context of this appendix, as the primary focus is on analyzing the impact of interventions on the spread of EVD. The intent of the presented model simulations is not to evaluate the model's performance against independent datasets but rather to investigate the potential outcomes of various intervention strategies. Future work may involve a comprehensive model evaluation to validate the predictions against real-world data.

### S3: Sensitivity

In the sensitivity analysis of our model, we investigated the effects of varying the recovery rate across a range of 0.067 to 0.256, divided into 23 equidistant values, and the basic reproductive number from 1.50 to 2.50, divided into 11 equidistant values. The analysis revealed that while the recovery rate predominantly influences the shape of the epidemic curve and the timing of the peak incidence, it does not significantly alter the cumulative case count. Conversely, fluctuations in R0 significantly impact both the cumulative number of cases and the peak incidence, underscoring the paramount importance of R0 as a determinant in the spread and severity of the EVD outbreak (Figure S4).


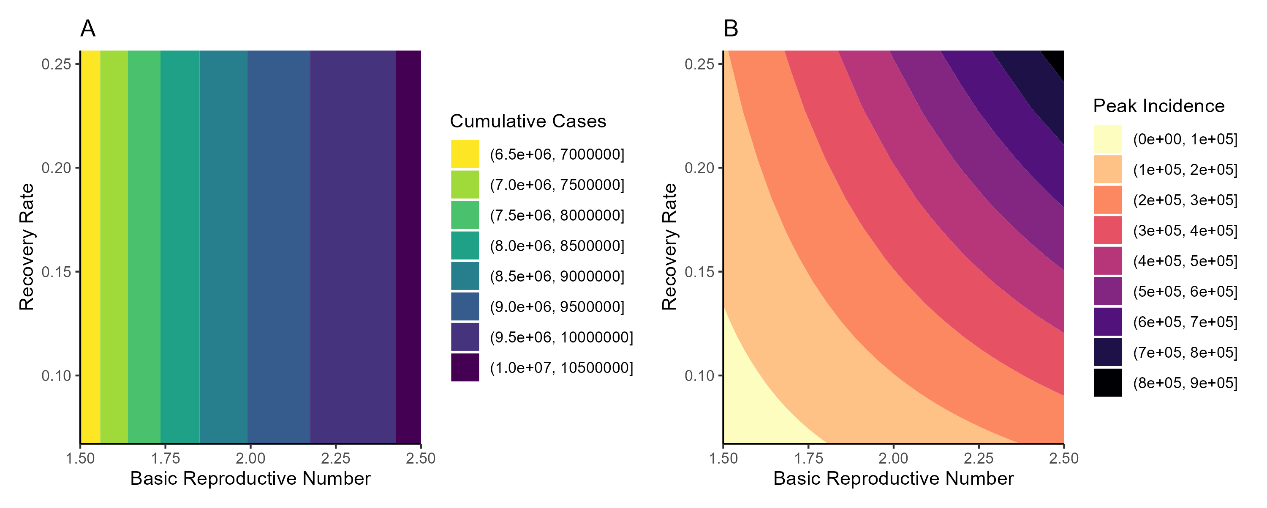


Figure S4: Simulated cumulative cases of EVD for different basic reproduction numbers and recovery rate.

# References

1. Velásquez GE, Aibana O, Ling EJ, et al. Time From Infection to Disease and Infectiousness for Ebola Virus Dise ase, a Systematic Review. *Clinical infectious diseases : an official publication of the Infectio us Diseases Society of America*;61(7):1135-40. doi: 10.1093/cid/civ531

2. Althaus CL. Estimating the Reproduction Number of Ebola Virus (EBOV) During the 20 14 Outbreak in West Africa. *PLoS Curr*;6:ecurrents.outbreaks.91afb5e0f279e7f29e7056095255b288. doi: 10.1371/currents.outbreaks.91afb5e0f279e7f29e7056095255b288

3. CDC. Treatment | Ebola (Ebola Virus Disease) | CDC 2022 [updated 2022/12/07/. Available from: <https://www.cdc.gov/vhf/ebola/treatment/index.htmlCDC>.

4. Senga M, Koi A, Moses L, et al. Contact tracing performance during the Ebola virus disease outbreak in Kenema district, Sierra Leone. *Philos Trans R Soc Lond B Biol Sci*;372(1721):20160300. doi: 10.1098/rstb.2016.0300

5. CDC. Guidance for Safe Handling of Human Remains of Ebola Patients in U. S. Hospitals and Mortuaries | Ebola Virus Disease | Clinicians | Ebola (Ebola Virus Disease) | CDC: CDC; 2023 [updated 2023/03/29/. Available from: <https://www.cdc.gov/vhf/ebola/clinicians/evd/handling-human-remains.html>.

6. Frimpong SO, Paintsil E. Community engagement in Ebola outbreaks in sub-Saharan Africa and implications for COVID-19 control: A scoping review. *Int J Infect Dis* 2023;126:182-92. doi: 10.1016/j.ijid.2022.11.032 [published Online First: 2022/12/04]

7. kaggle. Ebola | 2014-2016 | Western Africa Ebola Outbreak 2024 [updated 2024/01/19/. Available from: <https://www.kaggle.com/datasets/imdevskp/ebola-outbreak-20142016-complete-dataset/data>.

8. Bank W. World Bank Open Data 2024 [updated 2024/01/19/. Available from: <https://data.worldbank.org/indicator/SP.POP.TOTL?end=2016&locations=GN&most_recent_year_desc=true&start=2013>.

9. Dahl BA, Kinzer MH, Raghunathan PL, et al. CDC's Response to the 2014-2016 Ebola Epidemic - Guinea, Liberia, and Sierra Leone. *MMWR Suppl*;65(3):12-20. doi: 10.15585/mmwr.su6503a3

10. Parsamanesh M, Erfanian M, Mehrshad S. Stability and bifurcations in a discrete-time epidemic model with vaccination and vital dynamics. *BMC Bioinf* 2020;21(1):1–15. doi: 10.1186/s12859-020-03839-1

11. Wang B, Zou X, Zhu J. Data assimilation and its applications. *Proc Natl Acad Sci USA* 2000;97(21):11143–44. doi: 10.1073/pnas.97.21.11143

12. Katzfuss M, Stroud JR, Wikle CK. Understanding the Ensemble Kalman Filter. *American Statistician* 2016;70(4):350–57. doi: 10.1080/00031305.2016.1141709

13. Li K, Wang J, Xie J, et al. Advancements in Defining and Estimating the Reproduction Number in Inf ectious Disease Epidemiology. *China CDC weekly*;5(37):829-34. doi: 10.46234/ccdcw2023.158

# Figure legend

Figure S1: Flowchart of the susceptible-infected-recovered (SIR) model.

Figure S2: Cumulative cases of EVD.

Figure S3: Daily incidence of EVD.

Figure S4: Simulated cumulative cases of EVD for different basic reproduction numbers and recovery rate.
